# Injury induced expression of caveolar proteins in human kidney tubules - role of megakaryoblastic leukemia 1

**DOI:** 10.1186/s12882-017-0738-8

**Published:** 2017-10-24

**Authors:** Krzysztof M. Krawczyk, Jennifer Hansson, Helén Nilsson, Katarzyna K. Krawczyk, Karl Swärd, Martin E. Johansson

**Affiliations:** 1 Department of Translational Medicine, Clinical Pathology, Lund University, SUS Malmö, Jan Waldenströms gata 59, SE-20502 Malmö, Sweden; 2 Department of Laboratory Medicine, Lund University, Lund, Sweden; 3 Department of Experimental Medical Science, Lund University, Lund, Sweden

**Keywords:** Caveolae, Caveolin, Cavin, Kidney fibrosis, MKL1, FLNA, SCAI

## Abstract

**Background:**

Caveolae are membrane invaginations measuring 50–100 nm. These organelles, composed of caveolin and cavin proteins, are important for cellular signaling and survival. Caveolae play incompletely defined roles in human kidneys. Induction of caveolin-1/CAV1 in diseased tubules has been described previously, but the responsible mechanism remains to be defined.

**Methods:**

Healthy and atrophying human kidneys were stained for caveolar proteins, (caveolin 1–3 and cavin 1–4) and examined by electron microscopy. Induction of caveolar proteins was studied in isolated proximal tubules and primary renal epithelial cells. These cells were challenged with hypoxia or H_2_O_2_. Primary tubular cells were also subjected to viral overexpression of megakaryoblastic leukemia 1 (MKL1) and MKL1 inhibition by the MKL1 inhibitor CCG-1423. Putative coregulators of MKL1 activity were investigated by Western blotting for suppressor of cancer cell invasion (SCAI) and filamin A (FLNA). Finally, correlative bioinformatic studies of mRNA expression of caveolar proteins and MKL1 were performed.

**Results:**

In healthy kidneys, caveolar proteins were expressed by the parietal epithelial cells (PECs) of Bowman’s capsule, endothelial cells and vascular smooth muscle. Electron microscopy confirmed caveolae in the PECs. No expression was seen in proximal tubules. In contrast, caveolar proteins were expressed in proximal tubules undergoing atrophy. Caveolar proteins were also induced in cultures of primary epithelial tubular cells. Expression was not enhanced by hypoxia or free radical stress (H_2_O_2_), but proved sensitive to inhibition of MKL1. Viral overexpression of MKL1 induced caveolin-1/CAV1, caveolin-2/CAV2 and SDPR/CAVIN2. In kidney tissue, the mRNA level of MKL1 correlated with the mRNA levels for caveolin-1/CAV1, caveolin-2/CAV2 and the archetypal MKL1 target tenascin C (TNC), as did the MKL1 coactivator FLNA. Costaining for TNC as readout for MKL1 activity demonstrated overlap with caveolin-1/CAV1 expression in PECs as well as in atrophic segments of proximal tubules.

**Conclusions:**

Our findings support the view that MKL1 contributes to the expression of caveolar proteins in healthy kidneys and orchestrates the induction of tubular caveolar proteins in renal injury.

**Electronic supplementary material:**

The online version of this article (10.1186/s12882-017-0738-8) contains supplementary material, which is available to authorized users.

## Background

The human kidney is target for a vast array of disorders and kidney disease is increasing at an almost epidemic rate, imparting an enormous burden on patients and health care budgets. If the reparative mechanisms of the kidney are overrun, manifest organ injury will ensue, but the cellular source for renal epithelial regeneration has yet to be established unequivocally. The classical view that randomly surviving cells dedifferentiate and repopulate the injured tubules has recently been challenged by data favouring progenitor or stem cells as primary regenerative actors [1]. Regardless of this debate, we and others have described remarkable changes of tubular phenotype in response to injury and during regeneration, and also that the parietal epithelial cells (PEC) of Bowman’s capsule normally express the markers of these regenerating cells [2]. These tubular phenotypic alterations play an ill-defined role in disease resolution or progression. A protein that has consistently been found to become induced in the injured proximal tubular epithelium is caveolin-1/CAV1 [3–7]. Given that caveolin-1/CAV1 and caveolae have been proposed to influence signaling, transport and disease processes in the kidney [8–10] it is important to understand the mechanisms that underlie induction of caveolin-1/CAV1 and potentially other caveolar proteins in kidney disease.

Caveolae are 50–100 nm-sized membrane invaginations that play multiple roles in cell signaling and cholesterol homeostasis. They are present at high density in mesenchymal tissues such as striated and smooth muscle cells, adipocytes and in endothelial cells. Biogenesis of caveolae is a complex process, involving at least seven different genes from two families [11]. Most well-studied are the caveolins (caveolin-1/CAV1, caveolin-2/CAV2, caveolin-3/CAV3), which are integral membrane proteins with an unusual topology, where the N- and C-termini are intracellular. Recently discovered additional components of caveolae are the four cavin isoforms: Polymerase I and transcript release factor (PTRF/CAVIN1) Serum deprivation response (SDPR/CAVIN2), Protein kinase C delta binding protein (PRKCDBP/CAVIN3), and Muscle-related coiled-coil protein (MURC/CAVIN4). Cavins are cytosolic proteins that form homo- and heterotrimers that constitute building blocks for the striated coat of caveolae [11]. Rare null mutations in the genes responsible for caveolae result in lipo- and muscular dystrophies [12–14]. In addition, homozygous loss of PTRF/CAVIN1 has effects on smooth muscle motility and cardiac rhythmicity [14, 15]. A number of transcriptional control mechanisms for caveolin-1/CAV1 and expression of caveolae have been described. Among those with a positive regulatory impact are the myocardin family coactivators [16], hypoxia-inducible factor 1α (HIF1α) [17], peroxisome proliferator-activated receptor gamma (PPARG) [18], sterol regulatory element-binding proteins (SREBP) [19, 20] and forkhead box O (FOXO) transcription factors [21]. Any one of these transcription factors could tentatively be mechanistically responsible for de novo expression of caveolar proteins in diseased kidney tubules. Free radicals have moreover been proposed to induce caveolae, but the underlying mechanisms have not been precisely defined [22].

The myocardin family of transcriptional coactivators consists of four proteins: myocardin (MYOCD), megakaryoblastic leukemia 1 (MKL1), megakaryoblastic leukemia 2 (MKL2) and MEF2 activating motif and SAP domain containing transcriptional regulator (MAMSTR). These proteins stimulate transcription and play essential roles in striated and smooth muscle [23, 24]. The term “coactivator” reflects that myocardin family members bind to DNA via other transcription factors. The best established example is through the serum response factor (SRF) and via DNA motifs referred to as CArG-boxes [23, 24], but the cardiac isoform of myocardin may also influence transcription via MEF2 [25]. MKL1 (Megakaryoblastic leukemia 1) moreover interacts with SMAD3 and this complex binds to a GCCG-like motif in the human Slug promoter [26]. The latter effect is of relevance in the context of epithelial to mesenchymal transition. All myocardin family members have a conserved SAP domain (from SAF-A and -B/Acinus/PIAS) and a number of transcriptional targets appear to be SAP domain-dependent [27]. How the SAP domain binds to DNA however remains unclear. Studies have identified important roles of myocardin family members in cardiovascular [28, 29] and fibrotic [30, 31] diseases, and it is becoming increasingly apparent that the pro-motile and pro-contractile effects of these coactivators are important for the metastatic process [32, 33]. In cancer cells, it has been shown that MKL1 activity is strongly enhanced by down-regulation of its repressor SCAI (suppressor of cancer cell invasion) [32]. MKL1 signaling is also prevented by binding to the globular form of actin (G-actin), whereas polymerization into filamentous actin (F-actin) dislocates MKL1, thereby inducing nuclear translocation and activation of MKL1 [23]. Among the known regulatory factors positively influencing MKL1 is filamin A (FLNA). This is a structural protein that cross-links F-actin, attaches actin to integrins and also serves to anchor a set of plasma membrane proteins. FLNA acts through direct complex formation with MKL1 in the nucleus thereby enhancing transcription [34].

Recent experimental findings have defined roles for MKL1 and MKL2 in kidney cells. Work with kidney epithelial cell lines showed that disruption of intercellular junctions caused nuclear translocation of SRF and MKL2 and resultant transcriptional activation [35]. A recent study also showed that MKL1 regulates fibrosis in diabetic nephropathy [36].

The aim of the present work was to investigate the molecular mechanisms responsible for induction of caveolins and cavins in injured proximal tubules in situ and in tubular epithelial cells in vitro. We investigated the influence of hypoxia, free radicals and the myocardin family coactivator MKL1. Our studies support the view that MKL1 is responsible for the caveolin distribution in healthy and sclerotic human kidneys and for the induction of caveolar proteins in kidney epithelial cells in vitro. We furthermore present correlative evidence suggesting that SCAI repression and FLNA induction may contribute to MKL1 induced transcription and expression of caveolar proteins in tubular epithelial cells.

## Methods

### Immunohistochemistry

Tissues were fixed in 4% neutral buffered paraformaldehyde, paraffin embedded and cut at 3 μm. Immunohistochemistry was performed as described previously [2]. Staining was performed using the EnVision system for detection and DAKO Techmate 500 equipment, according to the instructions of the manufacturer (DAKO, Glostrup, Denmark). Chromogen was diaminobenzidine (DAB). For double stainings the Ventana Benchmark Ultra automated staining equipment was used according to routine protocols provided by the manufacturer. As chromogens, prefilled cartridges of OptiView DAB IHC Detection Kit and Alkaline Phosphatase Red (Ventana Medical Systems, Tucson, AZ) were used. The antibodies used were directed against caveolin-1/CAV1 (D46G3, 1:800, Cell Signaling, Danvers, MA), caveolin-2/CAV2 (610685), 1:500) and caveolin-3/CAV3 (610421, 1:500) both BD Transduction Laboratories, PTRF/CAVIN1 (ab78553, 1:500, Abcam, Cambridge, UK), SDPR/CAVIN2 (AF5759, 1:2000, R&D Systems, Abingdon, UK), PRKCDBP/CAVIN3 (16250–1-AP, 1:500, Proteintech, Manchester, UK) and TNC (MB1, 1:50, Novocastra, Leica Biosystems, Newcastle, UK). Hematoxylin was used as nuclear counterstain. As negative control, kidney tissue was stained according to the same protocol with exclusion of the primary antibody incubation step (Additional file 1: Figure S1).

### Transmission electron microscopy

Human kidney tissue was fixed in 2% glutaraldehyde buffered with 0.1 M cacodylate/0.1 M sucrose buffer, at pH 7.2. After 60 min the tissue was osmium treated, dehydrated and embedded in Agar 100 resin. Curing was performed at 60 °C for 48 h. Ultrathin sections were cut and stained with 2% uranyl acetate for 25 min and lead citrate for 2 min. TEM was performed using a JEOL 1230 microscope (Jeol, Tokyo, Japan) and 60 K digital images were captured. Caveolar diameters were determined using ImageJ (NIH, Bethesda, MD). Dimensions of caveolae in PECs were compared with caveolae in an existing image archive from human detrusor smooth muscle [37].

### Preparation of kidney epithelial cells and isolated tubules

Ethical permission was granted by the ethical committee at Lund University (LU680–08 and LU289–07) and the procedures followed were in accordance with the Helsinki Declaration. For isolation of primary kidney epithelial cells, kidneys were obtained from nephrectomies carried out due to localized renal tumors with written informed consent. Cortical tissue distant from the tumor was isolated and placed in cold Dulbecco’s modified Eagle’s medium (GE Healthcare, Logan, UT) with 10% fetal calf serum and 1% penicillin/streptomycin (Thermo Scientific, Waltham, MA). The tissue was rinsed, minced and subjected to overnight treatment (37 °C) with collagenase I (300 U/ml, Thermo Fischer Scientific Waltham, MA) and deoxyribonuclease I type II (200 U/ml, Sigma-Aldrich, St. Louis, MO). After trituration with a 10-ml pipette, the suspension was serially passed through tissue strainers with mesh sizes of 100 and 70 μm, excluding glomeruli. The suspension was thereafter treated with trypsin–EDTA for 5 min and passed through a 20-μm strainer, resulting in single cells.

For isolation of proximal tubules, cortical tissue was placed in DMEM medium supplemented with 10% fetal bovine serum (Saveen-Werner, Limhamn, Sweden) and 1% penicillin/streptomycin solution. One mm thick, cortical sections were prepared and incubated in 250 U/ml collagenase type II (Invitrogen) solution with shaking to digest the tissue. Proximal and distal tubules were isolated manually using watchmakers forceps and a standard cell culture inverted microscope under ocular inspection. Tubules were isolated according to their varying thickness, refractive appearance and degree of convolution. The isolation protocol was validated by immunohistochemistry and lectin staining. Positive markers for proximal tubules were: CD10 (Ventana, Tuscon, AZ) and Fluorescein labeled *Lotus tetragonolobus* Lectin (Vector Laboratories, Burlingame, CA). Positive markers for distal tubules were E-cadherin (Ventana, Tuscon, AZ) and *Dolichos biflorus* Agglutinin (Vector Laboratories, Burlingame, CA). Results from the validation experiments for isolation of proximal and distal tubules are shown in Additional file 2: Figure S2. The tubular contamination was negligible. Cell cultures were initiated from either isolated tubules or digested cortical tissue.

Primary kidney epithelial cells and cells from isolated proximal tubules were cultured in DMEM high glucose supplemented with 10% fetal bovine serum and 1% penicillin/streptomycin solution, at 37 °C and 5% CO_2_. Cell culture experiments were also conducted using serum free medium [38] Briefly, for these experiments DMEM/F12 medium (GE Healthcare, South Logan, UT) supplemented with insulin transferrin selenium (Thermo Fischer Scientific, Waltham, MA), 10 ng/ml of epidermal growth factor (Thermo Fischer Scientific, Waltham, MA), 10 pg/ml of triiodothyronine (Sigma Aldrich, St Louis, MO), 36 ng/ml hydrocortisone (Sigma Aldrich, St Louis, MO) and 1% penicillin/streptomycin solution was used. Cells were cultured until they reached 50% confluency, 100% confluency, or until 3 or 5 days post confluency.

### Treatment protocols

To induce oxidative stress, cells were treated with 700 μM H_2_O_2_ (Sigma Aldrich, St Louis, MO) for 2 h. Thereafter medium was exchanged and cells were left to recover in the incubator for 24 h, 48 h and 72 h.

Hypoxia was generated in a Whitley H35 Hypoxystation (Don Whitley Scientific, Shipely, UK). Primary renal cell cultures were placed in the hypoxia chamber at 1% pO_2_ and medium was changed to hypoxic pre-conditioned media. Cells were incubated for 24 h and 72 h. Control cells were incubated in 21% pO_2_.

To inhibit MKL1, cells were treated with CCG-1423 (Sigma Aldrich, St Louis, MO). Cells exposed to the inhibitor were harvested at 50% confluency, 100% confluency, and at 3 and 5 days post confluency. At each time point, control and treated cells were collected for Western blot analysis. Cells were scraped in ice cold PBS, spun down and snap frozen in liquid nitrogen. Pellets were stored in −80 °C until further analysis.

For viral transduction, kidney epithelial cells were seeded in 6-well plates. At 24 h after seeding, cells were transduced using 25, 50 and 100 MOI (multiplicity of infection) of Ad-CMV-MKL1-eGFP (Vector Biolabs Burlingame, CA). As a control 25, 50 and 100 MOI respectively of Ad-CMV-null was used. Cells were incubated with virus for 96 h and then harvested for qPCR analysis.

### Quantitative PCR

Nucleic acid was extracted using the QIAshredder and RNeasy Mini Plus kit (Qiagen Inc., Hilden, Germany). The quality and concentration of RNA was determined using a Nanodrop spectrophotometer (Thermo Fischer Scientific, Waltham, MA). cDNA was synthesized with random primers and reverse transcriptase enzyme (MultiScribe, Applied Biosystems, Foster City, CA). The GeneAmp 7300 sequence detector was used for qPCR with the SYBR Green Master Mix (Applied Biosystems, Foster City, CA). Reactions were performed in triplicate, and three housekeeping genes (UBC, YWHAZ, and SDHA) were used for normalization (Table 1).

**Table 1:** Primer sequences used in the analysis

Gene	Forward (5′-3′)	Reverse (5′-3′)
UBC	5′-ATTTGGGTCGCGGTTCTTG	5′-TGCCTTGACATTCTCGATGGT
YWHAZ	5′-ACTTTTGGTACATTGTGGCTTCAA	5′-CCGCCAGGAGGACAAACCAGTAT
SDHA	5′-TGGGAACAAGAGGGCCATCTG	5′-CCACCACTGCATCAAATTCATG
CAV1	5′-GAAAGAAGATGGGGGAGGAG	5′- AAAGTCCCCAAAGGCAGAAT
CAV2	5′-ACGACTCCTACAGCCACCAC	5′-CGTCCTACGCTCGTACACAA
CAVIN1	5′-GCTCCTTCCGAACTTCCTCT	5′-ACTTGGACAACCAGGACAGG
CAVIN2	5′-CTTGTGCCTTGTCCCAAAAT	5′-CGCGTAGCTACCCTCATAGC
CAVIN3	5′- CTTGTGCCTTGTCCCAAAAT	5′- TTATTGATGGTGAGCGCAAG

For the qPCR results in Fig. 6, cells were lysed in Qiazol and total RNA was isolated with Qiagen miRNeasy mini kit in a Qiacube according to the manufacturer’s instructions (Qiagen, Hilden, Germany). qPCR reactions were performed on a real time thermal cycler (StepOnePlus™, Applied Biosystems, Foster City, CA) using Quantifast SYBR Green RT-PCR kit and Quantitect primer assays for: 18S, caveolin-1/CAV1, caveolin-2/CAV2, PTRF/CAVIN1 and SDPR/CAVIN2. The comparative Ct method (2–[delta][delta]Ct method) was used to quantify relative RNA levels for all qPCR experiments.

### Western blotting

Frozen cell pellets were lysed with RIPA buffer containing cOmplete™ protease inhibitor cocktail (Sigma Aldrich, St Louis MO) for 20 min on ice. Every five minutes, lysates were vortexed. On completion of lysis, lysates were spun for 20 min at 4 °C to pellet remaining debris. Protein lysates from human kidney cortex were prepared by disrupting the tissue using a TissueLyser LT (Qiagen, Hilden, Germany) for two cycles of 30 s at 50 Hz in RIPA buffer. Lysates were further disrupted by sonication and spun for 20 min at 4°C to remove debris. Protein concentrations were determined using the Bradford assay. Western blotting was performed by standard methods using precast gels and the Trans-blot Turbo transfer system from BIO-RAD, (Hercules, CA). The following primary antibodies were used: caveolin-1/CAV1 (D46G3) from Cell Signaling (Danvers, MA), caveolin-2/CAV2 (610685) and HSP90 (610418) from BD Transduction Laboratories (San Jose, CA), PRKCDBP/CAVIN3 (16250–1-AP) from Proteintech (Rosemont, IL), β-actin (A5441) from Sigma-Aldrich (St Louis, MO) and PTRF/CAVIN1 (ab48824), SDPR/CAVIN2 (ab113876), PRX-SO_3_ (ab16830), Filamin A (ab76289) and SCAI (ab124688) from Abcam (Cambridge, UK). HRP-conjugated anti-mouse and anti-rabbit secondary antibodies from Cell Signaling (Danvers, MA) were used and bands were visualized using enhanced chemiluminescence (Pierce West Femto, Thermo Fischer Scientific, Waltham, MA) in an Odyssey Fc Imager (LI-COR Biosciences, Lincoln, NE).

### Statistical analyses

Kidney mRNA expression data (*n* = 32) were retrieved from the GTEx project (https://gtexportal.org/home/) [39] and normalized using the method of Robinson and Oshlack [40]. Correlation coefficients were calculated using the Spearman method in Graph Pad Prism (GraphPad Software, La Jolla, CA). *P* < 0.05 was considered significant. For the remainder of our experiments, pair-wise comparisons were made using an unpaired Student’s t-test. Multiple comparisons were done using one-way ANOVA followed by the Bonferroni post-hoc test.

## Results

### Investigation of the tissue distribution of caveolar proteins in human kidney

**Fig. 1:**
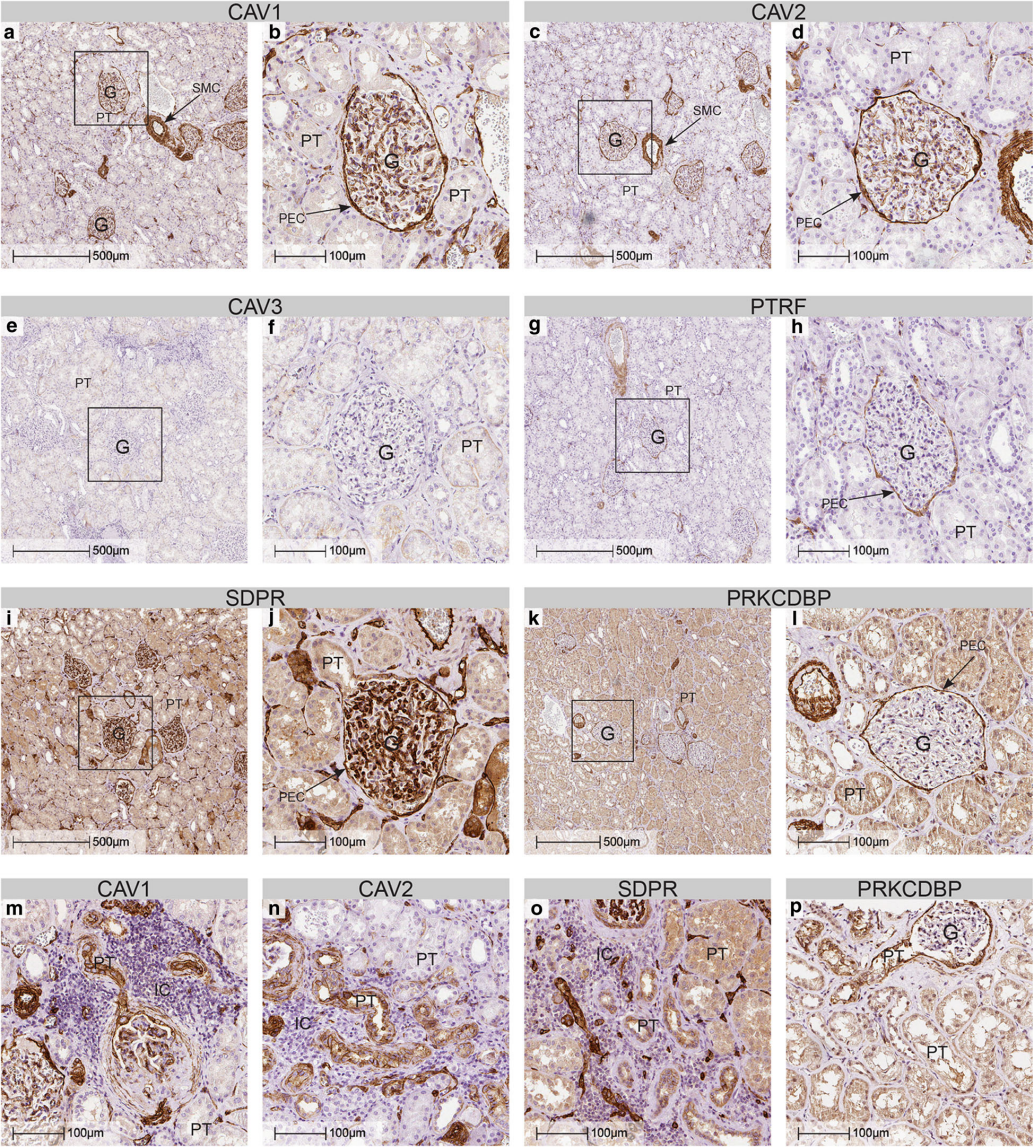
Caveolar proteins are normally not expressed in proximal tubules, but are induced in atrophic tubular segments. Immunohistochemical stainings of human renal tissue showing the distribution of caveolar proteins. Expression of caveolin-1/CAV1 and caveolin-2/CAV2 (**a** through **d**) was observed in the PECs, endothelial cells and smooth muscle cells (SMC). No caveolin-3/CAV3 staining could be detected (**e** and **f**). PTRF/CAVIN1 staining was observed in PECs and SMCs of large arteries (**g** and **h**). PECs and endothelial cells stained positive for SDPR/CAVIN2 (**i** and **j**). PRKCDBP/CAVIN3 staining was observed in PECs, and SMCs of arteries, (**k** and **l**) whereas MURC/CAVIN4 staining could not be detected (data not shown). In proximal tubules undergoing atrophy due to normal kidney ageing, tubular cells were positive for caveolin-1/CAV1, caveolin-2/CAV2, SDPR/CAVIN2 and PRKCDBP/CAVIN3 (**m**-**p**). G - glomerulus, PT - proximal tubules, IC - inflammatory cells, PEC - parietal epithelial cells. Inserted squares mark areas magnified in the following image

In order to determine the cell-type specific expression of caveolar proteins in kidney tissue, healthy human kidney samples were stained using antibodies against caveolins (caveolin-1/CAV1, caveolin-2/CAV2 and caveolin-3/CAV3) and cavins (PTRF/CAVIN1, SDPR/CAVIN2, PRKCDBP/CAVIN3 and MURC/CAVIN4). The results of the stainings are shown in Fig. 1 and summarized in Table 2. Smooth muscle cells expressed caveolin-1/CAV1, caveolin-2/CAV2, PTRF/CAVIN1 and PRKCDBP/CAVIN3 as expected. Caveolin-1/CAV1 staining was also seen in the endothelium of blood vessels, including glomerular and peritubular capillaries and in the PECs of Bowman’s capsule (Fig. 1a, b). The proximal tubules were however negative. Staining patterns for caveolin-2/CAV2, PTRF/CAVIN1, SDPR/CAVIN2 and PRKCDBP/CAVIN3 overlapped considerably with this pattern (Fig. 1c-d and g-l). In arteries however, SDPR/CAVIN2 appeared to be preferentially localized to endothelial cells whereas PRKCDBP/CAVIN3 was preferentially localized to the vascular media. Little, if any, staining for caveolin-3/CAV3 and MURC/CAVIN4 could be detected (Fig. 1e-f and data not shown). Thus, caveolar proteins were detected in normal kidney, but not in proximal tubules. Normal kidney tissue always contains focal sclerotic glomeruli, as part of the normal ageing process. In these areas, the adjoining proximal tubules showed light microscopic signs of atrophy, such as narrowing of diameter, basal membrane thickening and wrinkling of the basal membrane. Interestingly, in the proximal tubular segments adjacent to sclerosing glomeruli, we could detect staining for caveolin-1/CAV1, caveolin-2/CAV2, SDPR/CAVIN2 and PRKCDBP/CAVIN3 (Fig. 1m-p and Table 2). These results indicate that proximal tubular atrophy involves *de-novo* expression of caveolar proteins.

**Table 2:** Distribution of caveolin and cavin isoforms in human kidney tissue as assessed by immunohistochemistry

	Proximal tubules		PEC	Endothelial cells			SMC
	normal	sclerotic segments	normal	glomerular endothelium	capillary endothelium	larger arteries	
caveolin-1/CAV1	–	**+**	**+**	**+**	**+**	**+**	**+**
caveolin-2/CAV2	–	**+**	**+**	**+**	**+**	**+**	**+**
caveolin-3/CAV3	–	–	–	–	–	–	–
PTRF/CAVIN1	–	–	**+**	–	–	**+**	**+**
SDPR/CAVIN2	–	**+**	**+**	**+**	**+**	**+**	–
PRKCDBP/CAVIN3	–	**+**	**+**	–	–	**+**	**+**
MURC/CAVIN4	–	–	–	–	–	–	–

The symbols + and - denote presence and absence of staining respectively

### Electron microscopy and calculation of renal caveolar size distribution

**Fig. 2:**
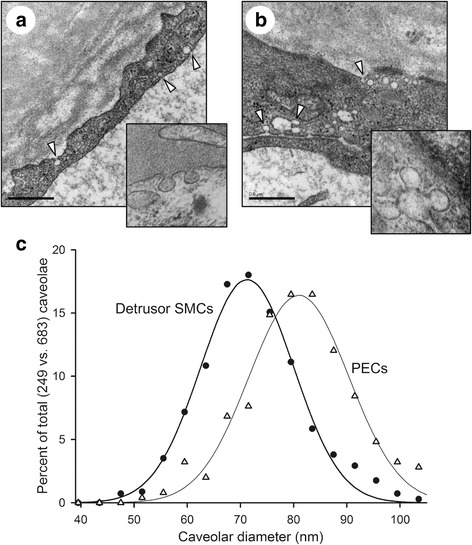
Parietal epithelial cells of Bowman’s capsule display a high density of caveolae as demonstrated by electron microscopy. Panels **a** and **b** show presence of caveolar invaginations in apical and basal plasma membranes of PECs, marked by arrowheads (insets show higher magnification views). Scale bar: 0,5 μm. Panel **c** shows the size distribution of caveolae in PECs as compared to human detrusor smooth muscle cells, showing a median size of 80 nm

To ascertain that positive staining for caveolar proteins corresponds to morphologically identifiable caveolae, kidneys were examined by transmission electron microscopy. We focused on PECs in view of the intense staining for caveolar proteins in these cells. Caveolae were present at a relatively high density in PECs and they had both apical and basal orientations (Fig. 2a, b), confirming that positive staining for caveolar proteins indeed coincides with the presence of caveolae at the ultrastructural level.

Invaginated structures with several connected caveolae, so called rosettes, were also observed (Fig. 2b, insert). The size distribution of caveolae in PECs (Fig. 2c) suggested a somewhat larger size than in human detrusor cells, 80 vs 70 nm in mean diameter, but the range was clearly within limits reported for caveolae in other tissues. Caveolae were absent from epithelial cells in uninjured proximal tubules (not shown).

### Exposure to H_2_O_2_ or hypoxia does not induce the expression of caveolar proteins in primary kidney epithelial cells

**Fig. 3:**
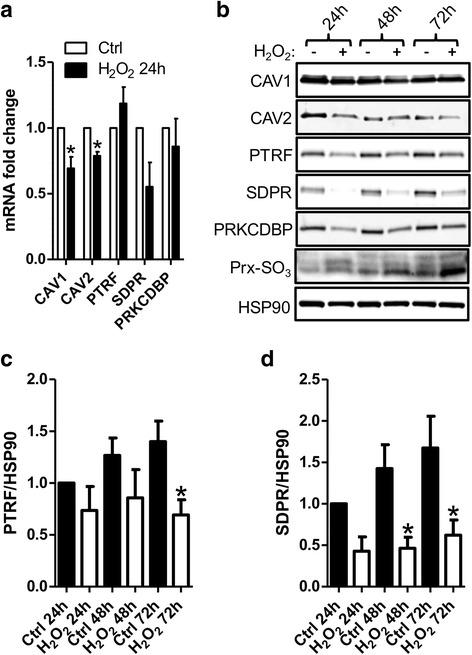
Suppression of caveolins and cavins by H_2_O_2_ in primary kidney epithelial cells. To explore putative causative mechanisms for the induction of caveolar mRNA and proteins in sclerotic kidneys, primary renal epithelial cells were cultured in vitro with and without H_2_O_2_ exposure. The mRNA levels were analyzed by qPCR at 24 h (**a**) and by Western blotting at 24-72 h (**b-d**). **c** and **d** show the quantification of Western blot results for PTRF/CAVIN1 and SDPR/CAVIN2, *(*p* < 0.05), *n* = 6. Induction of oxidative stress was confirmed by increased oxidation of Prx-SO_3_

In an attempt to understand the basis for tubular epithelial induction of caveolar proteins in sclerotic kidneys we next tested plausible triggering mechanisms using primary kidney epithelial cells in culture. Previous studies in other systems have shown that free radicals may induce expression of caveolins and caveolae [22]. Furthermore, oxidative stress is a likely insult causing tubular atrophy. However, when kidney epithelial cells were exposed to H_2_O_2_ we found that the mRNA levels for renal caveolin and cavin isoforms were unchanged or even reduced (Fig. 3a). Western blotting for caveolins and cavins similarly revealed reductions of caveolin-2/CAV2, PTRF/CAVIN1, SDPR/CAVIN2 and PRKCDBP/CAVIN3 (Fig. 3b-d) whereas caveolin-1/CAV1 protein levels remained unchanged (Fig. 3b). The oxidation of the enzyme peroxiredoxin (Prx-SO_3_), a measure of oxidative stress, increased as expected, confirming oxidative stress (Fig. 3b bottom). These findings did therefore not support increased oxidative stress as an explanation for the induction of caveolar proteins seen in atrophic tubules.

**Fig. 4:**
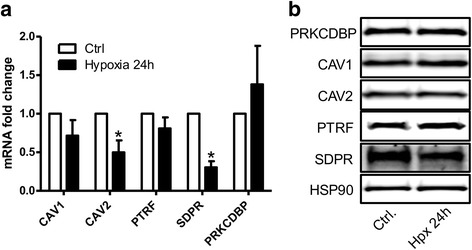
Hypoxia has an inhibitory effect on some of the caveolin and cavin levels. To investigate if hypoxia may induce expression of caveolar proteins, cultured tubular cells were exposed to hypoxia (1% O_2_). The mRNA (**a**) and protein levels (**b**) of caveolins (caveolin-1/CAV1, caveolin-2/CAV2) and cavins (PTRF/CAVIN1, SDPR/CAVIN2, PRKCDBP/CAVIN3) in primary kidney epithelial cells after normoxic (white bars) or hypoxic (black bars) culture for 24 h are shown. Hypoxia resulted in statistically significant decrease of caveolin-2/CAV2 and SDPR/CAVIN2 expression, whereas the other caveolar proteins were unaltered *(*p* < 0.05)

Hypoxia and hypoxia-inducible factor 1 have been demonstrated to positively regulate caveolin-1/CAV1 in clear cell renal cell carcinoma [17]. We therefore next addressed the possibility that hypoxia may be the causative factor for the induction of caveolar proteins in atrophic tubules. Contrary to expectation, after culturing primary kidney epithelial cells for 24 h at 1% oxygen the mRNA levels for caveolin-2/CAV2 and SDPR/CAVIN2 were reduced (Fig. 4a). SDPR/CAVIN2 was reduced also at the protein level whereas the other caveolins and cavins remained unchanged (Fig. 4b). The directionality of these changes argued against hypoxia as a causative mechanism for induction of caveolins and cavins in human kidney epithelial cells.

### Tubular microdissection as a means to study tubular caveolar induction

**Fig. 5:**
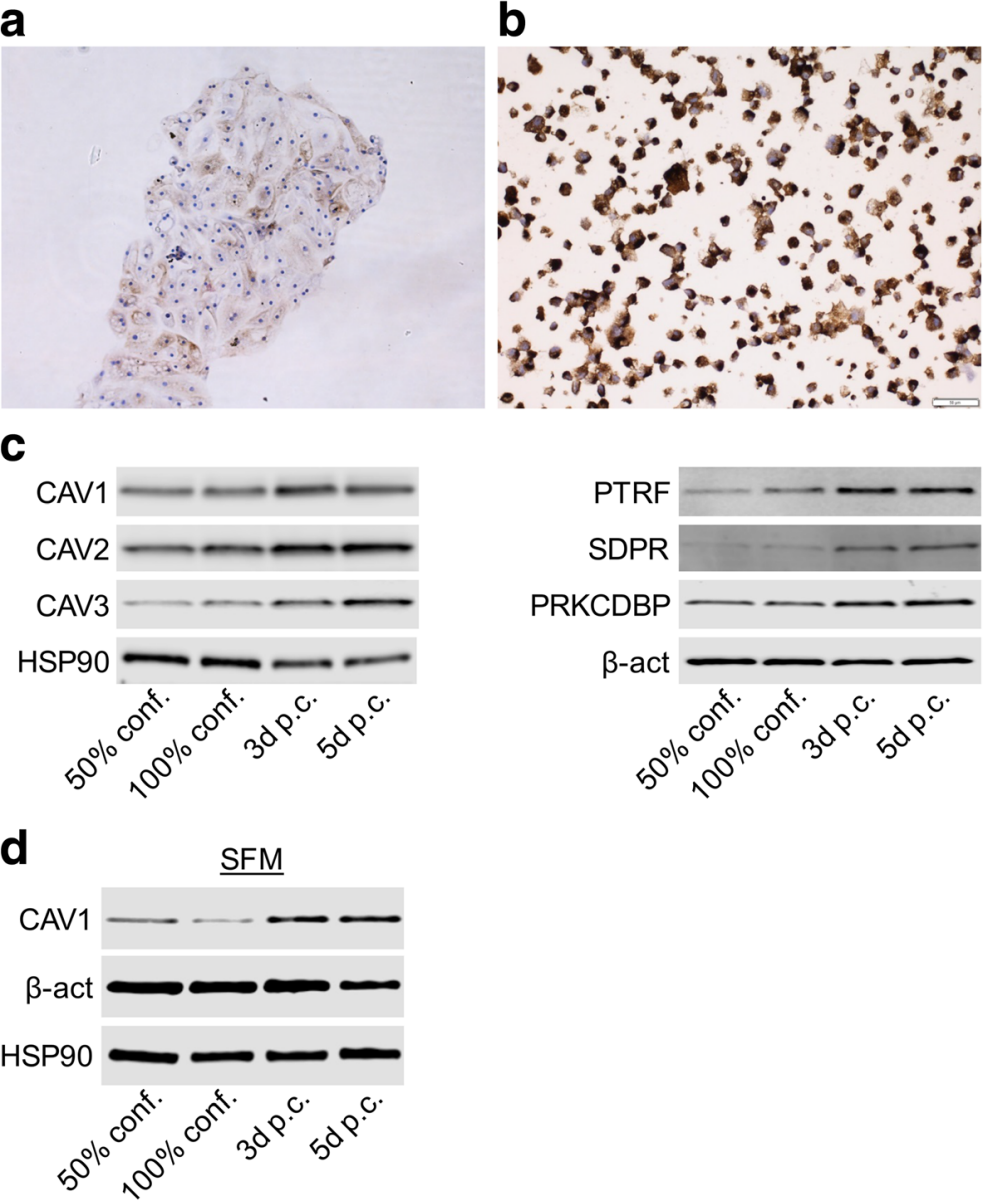
Spontaneous induction of caveolins and cavins by proximal tubule epithelial cell dissociation and culture. **a**. Immunohistochemical staining for caveolin-1/CAV1 in a manually isolated proximal tubule. Caveolin-1/CAV1 is positive in most cells growing out from the collapsed proximal tubule. **b**. Proximal tubules were manually isolated to homogeneity, enzymatically dissociated and cultured. Following formalin fixation, cells were stained for caveolin-1/CAV1. Only a minor fraction of colonies should have been positive for caveolin-1/CAV1 if cultures were overgrown by a caveolin-1/CAV1- positive progenitor cell, but instead we found all resulting colonies to be positive. The increased expression of caveolins (CAV1–3) and cavins (PTRF/CAVIN1, SDPR/CAVIN2 and PRKCDBP/CAVIN3) with time in culture are illustrated by Western blot in panel (**c**). Panel **d** presents the increased expression of caveolin-1/CAV1 when cultured in serum free medium (SFM). Quantification of Western blot data is shown in Additional file 3: Figure S3

We next reflected on the ease with which caveolins and cavins could be detected in primary kidney epithelial cells in culture, contrasting with the situation in situ where healthy proximal tubules were negative for these proteins. The kidney epithelial cells used were prepared by standard methods where the full kidney cortex is dissociated into single cell culture, after sieving the glomeruli. Even though the major part of the cortex is made up of proximal tubules it is formally possible that such cultures could be dominated by a cell type of other origin, such as PECs, which stained positive for caveolin-1/CAV1 and caveolin-2/CAV2 as well as cavin 1–3 in healthy kidneys. To approach this issue, we devised a microdissection strategy in which proximal tubules were manually separated from distal tubules and glomeruli. In cell culture conditions, isolated proximal tubules first adhered to the culture dishes followed by collapse of the tubular structure and attachment to the substrate. Cells then started to proliferate in all directions. We were able to detect positive staining for caveolin-1/CAV1 in a major fraction of the cells already at the stage of tubular collapse (Fig. 5a). We also enzymatically dissociated cells from batches of isolated proximal tubules, seeded them and allowed them to form colonies. When such cells were stained for caveolin-1/CAV1 they were uniformly positive (Fig. 5b). Having noted the early up-regulation of caveolin-1/CAV1 during tubular cell culture, we next set out to quantify the spontaneous induction of caveolins and cavins in culture by Western blotting. Cells were harvested at 50 and 100% confluence and at 3 and 5 days post-confluency. In keeping with the staining results from the isolated proximal tubules, we found steady induction of all caveolins and cavins reaching a maximum at 5 days post-confluence (Fig. 5c, summarized results in Additional file 3: Figure S3). Choice of medium may affect results from in vitro experiments. Therefore we cultured epithelial cells isolated using the standard protocol in a defined serum free medium (SFM). Similar results were obtained using this defined medium (Fig. 5d).

### Manipulation of caveolar proteins by MKL1 inhibition and overexpression

**Fig. 6:**
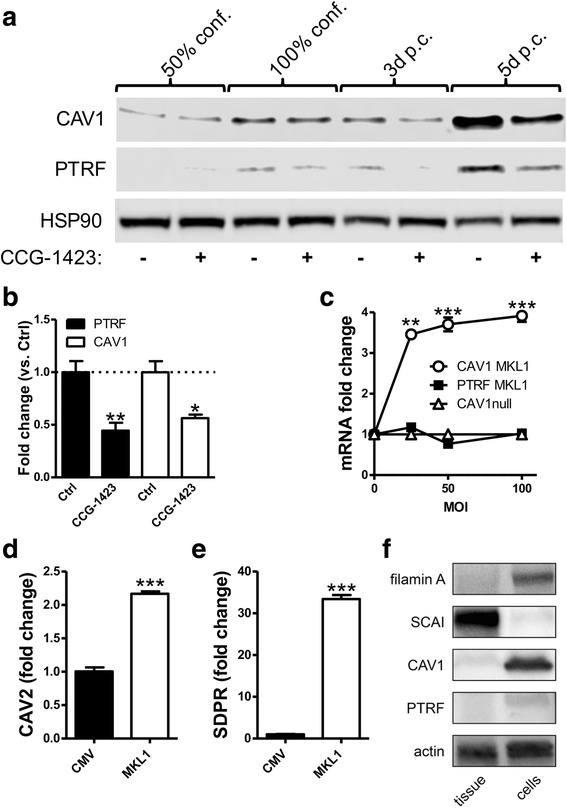
Control of caveolar proteins by megakaryoblastic leukemia 1 (MKL1). **a**. Western blots showing protein levels of caveolin-1/CAV1 and PTRF/CAVIN1 at different cell densities and at 3 and 5 days post-confluence in the absence (−) or presence (+) of 10 μM CCG-1423. Quantification of Western blot data from CCG-1423 treatment at 5 days post-confluence are shown in (**b**) (*n* = 4). **c**. Results of qPCR showing caveolin-1/CAV1 and PTRF/CAVIN1 mRNA levels after viral transduction with different multiplicities of infection (MOI) of Ad-CMV-MKL1. Panels **d** and **e** show qPCR for caveolin-2/CAV2 and SDPR/CAVIN2 at 100 MOI of Ad-CMV-null or Ad-CMV-MKL1. *(*p* < 0.05), **(*p* < 0.01), ***(*p* < 0.001). **f**. Western blots for the MKL1 coactivator FLNA and repressor SCAI, comparing levels in normal kidney cortical tissue lysates and cultured kidney epithelial cells obtained from the same kidneys 5 days post confluency. As caveolin-1/CAV1 and PTRF/CAVIN1 expression becomes evident during culture, a parallel de novo expression of FLNA is seen. Conversely, the pronounced cortical levels of SCAI are diminished during culture

It has recently been discovered that two members of the myocardin family of transcriptional coactivators (MKL1 and MYOCD) control the majority of genes involved in biogenesis of caveolae in smooth muscle. In unrelated studies one of the myocardin family members, MKL1 has been shown to be involved in epithelial-to-mesenchymal transition [41] and kidney fibrosis [36, 42]. We therefore considered the possibility that MKL1 may be responsible for the induction of caveolin-1/CAV1 and other caveolar proteins in atrophic proximal tubules and in tubular cells in primary culture. To address this, proximal tubular epithelial cells were treated with the MKL1 inhibitor CCG-1423 to test if this substance would inhibit the spontaneous induction of caveolar proteins in culture. In keeping with the hypothesis CCG-1423 reduced the protein levels of caveolin-1/CAV1 and PTRF/CAVIN1 significantly (Fig. 6a and b) at 5 days post-confluence. We next transduced MKL1 into kidney epithelial cultures using an adenovirus (Ad-CMV-MKL1) and examined the mRNA levels of CAV1–2, SDPR/CAVIN2 and PTRF/CAVIN1. Similar to the spontaneous induction seen during primary culture, MKL1 transduction increased caveolin-1/CAV1 mRNA levels, however not PTRF/CAVIN1 mRNA levels (Fig. 6c). Caveolin-2/CAV2 was also induced by MKL1 transduction (Fig. 6d), and SDPR/CAVIN2, whose spontaneous induction was largest of all caveolae proteins, increased more than 30-fold (Fig. 6e). Slight discrepancies between protein and mRNA levels for caveolins and cavins are to be expected, because formation of caveolae is known to stabilize caveolins and cavins at the protein level. Taken together, these findings supported the view that MKL1 may be responsible for the induction of caveolins and cavins in primary culture of kidney epithelial cells.

### Analysis of MKL1 repressing and enhancing cofactors in tubular cell culture

Our results so far demonstrated that caveolin-1/CAV1 and PTRF/CAVIN1 are absent from normal cortical tubules, but show increased levels during primary cell culture and that this increase is controlled by MKL1 activity. To further investigate the control of MKL1 activity we also analyzed the expression of the known MKL1-repressor SCAI and the MKL1 coactivator FLNA in protein lysates from normal kidney cortex and compared these with lysates from cultured tubular cells. We found SCAI to be expressed at high levels in normal kidney cortex, but absent in cultured tubular cells. The reverse pattern was observed for FLNA, which was strongly induced by culture of cortical epithelial tissue (Fig. 6f). We therefore suggest that MKL1 activity in cultured tubular epithelial cells may be affected by reciprocal changes in FLNA and SCAI.

### MKL1 correlation analysis using the GTEx data base and staining for the MKL1 target gene TNC in pathological kidney tissue

**Fig. 7:**
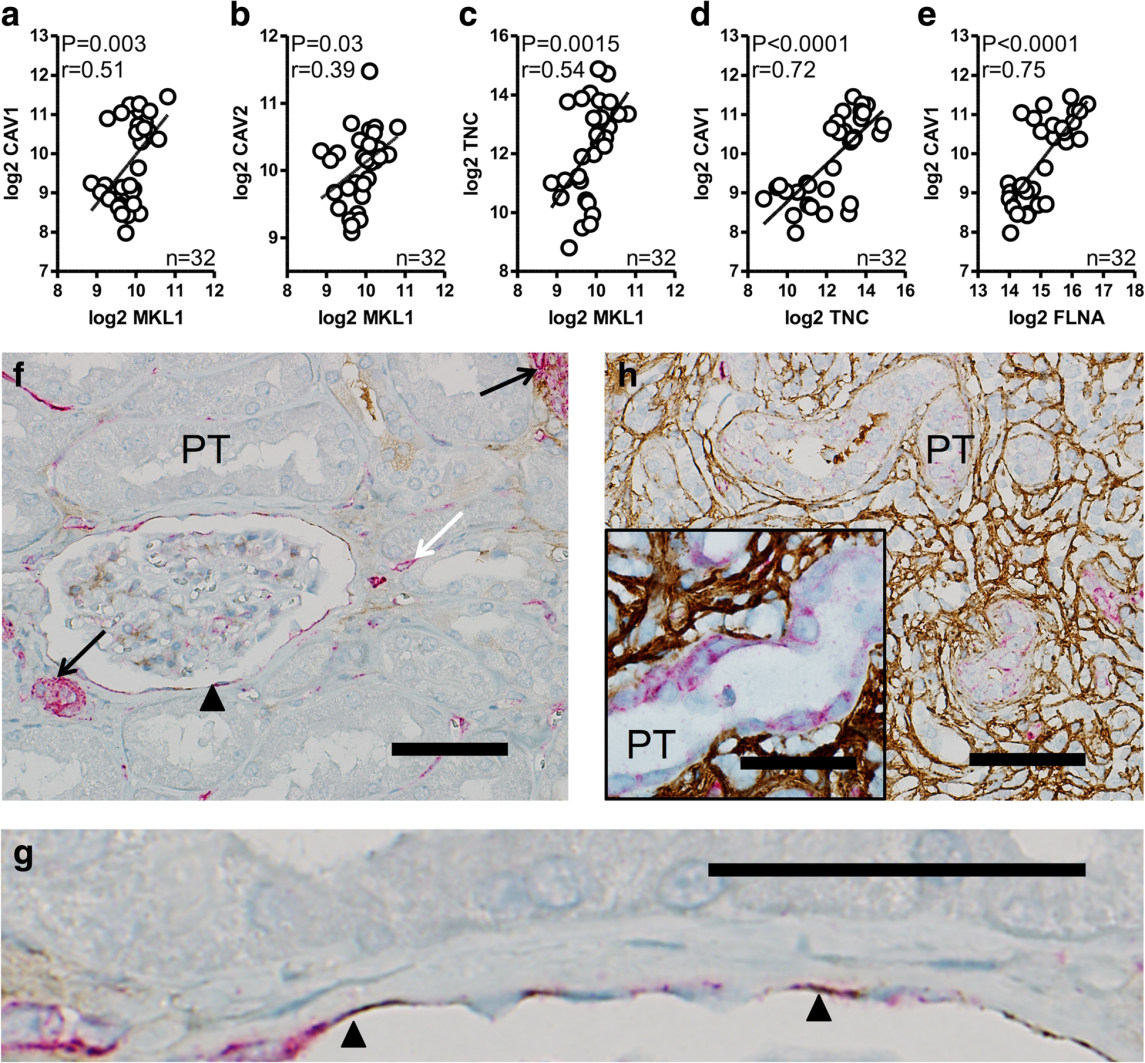
MKL1, TNC and FLNA correlation analysis using GTEx data and immunohistochemical colocalization of caveolin-1/CAV1 and TNC in healthy and diseased human kidneys. Human kidney expression data were retrieved from the GTExPortal and MKL1 expression levels were correlated with caveolin-1/CAV1 (**a**), caveolin-2/CAV2 (**b**) and TNC (**c**) using the Spearman test. Spearman Rho values and *P*-values are given in each panel. TNC (**d**) and FLNA (**e**) expression levels also correlated with caveolin-1/CAV1. Panels F through H show human kidney tissue co-stained for TNC (brown) and caveolin-1/CAV1 (red). In (**f**) and (**g**), staining of normal kidney tissue is shown. Caveolin-1/CAV1 expression is limited to PECs (black arrowhead), smooth muscle cells of the arterial media (black arrow) and endothelial cells (white arrow). PECs also co-express TNC as shown by the presence of both brown and red staining in panel G which shows Bowman’s capsule at high magnification with a distinct, but thin, linear basal TNC band. In **h**, kidney tissue demonstrating signs of tubular atrophy due to nephrosclerosis is shown. Here, a diffuse reticular and interstitial TNC deposition is seen (brown). Adjacent tubules show signs of atrophy in the form of epithelial simplification and loss of tubular width. Caveolin-1/CAV1 is expressed in the injured tubules. Caveolin-1/CAV1 positive (red) proximal tubular cells (PT) resting on a TNC-positive matrix (brown) are shown at higher magnification in the inset of panel (**h)**. All specimens were counterstained with hematoxylin. Scale bars in **f** and **h** = 100 μm. Scale bar of inset in **h** = 50 μm. Scale bar in **g** = 75 μm

To further explore whether MKL1 might be responsible for the expression of caveolar proteins in situ*,* we examined if correlations existed between MKL1 and CAV1/CAV2 using the expression database GTExPortal.com. The mRNA level of MKL1 in human kidney indeed correlated significantly with the levels of both caveolin-1/CAV1 (Fig. 7a) and caveolin-2/CAV2 (Fig. 7b). An archetypal target of MKL1 is tenascin C (TNC) [43], and as expected, MKL1 correlated significantly with TNC in human renal tissue (Fig. 7c). TNC, in turn, correlated with caveolin-1/CAV1 (Fig. 7d) and also the MKL1 coactivator FLNA showed strong correlation with caveolin-1/CAV1 (Fig. 7e). We next used TNC staining as a readout for MKL1 activity in kidney tissue. In healthy kidneys, PECs of Bowman’s capsule were found to rest on a thin rim of TNC-positive matrix (Fig. 7f, g), while proximal and distal tubules were negative for both TNC and caveolin-1/CAV1. In contrast, in sclerotic kidneys with distinctly atrophic tubules, caveolin-1/CAV1 positive proximal tubular epithelial cells were found to rest on a reticular matrix that stained strongly positive for TNC (Fig. 7h). Epithelial TNC staining therefore coincided with caveolin-1/CAV1 staining in healthy and diseased human kidneys, supporting the view that these proteins are co-regulated in kidney epithelial cells.

## Discussion

We find that caveolins (caveolin-1/CAV1, caveolin-2/CAV2, but not caveolin-3/CAV3) and cavins (PTRF/CAVIN1, SDPR/CAVIN2, and PRKCDBP/CAVIN3, but not MURC/CAVIN4) are highly expressed in blood vessels and in the PECs of Bowman’s capsule in healthy human kidney tissue, but absent from proximal tubules. In sclerotic kidneys on the other hand, caveolar proteins (caveolin-1/CAV1, caveolin-2/CAV2, SDPR/CAVIN2 and PRKCDBP/CAVIN3) are also expressed in atrophying proximal tubules. In vitro studies showed that caveolins and cavins are induced in epithelial cells in culture and that this can be inhibited by an MKL1 inhibitor and mimicked by viral overexpression of MKL1. We also show that CAV1/CAV2 mRNA expression levels correlate with MKL1 and with the archetypal MKL1 target tenascin C (TNC) in human kidney tissue. TNC and caveolin-1/CAV1 stainings coincide in both normal and diseased kidneys. Together these findings argue that MKL1 activity is an important determinant of caveolin-1/CAV1 distribution in healthy and diseased kidneys. It is interesting that PECs are endowed with such a broad repertoire of caveolar proteins, normally only encountered in mesenchymal cell types. To our knowledge this is clearly a unique feature of this epithelium. The stratum basale of stratified squamous epithelia, although structurally dissimilar, is the only other epithelial tissue that at least partially recapitulates this structural aspect.

Our findings are well in line with the recent finding that myocardin and MKL1 regulate caveolin and cavin expression in human coronary artery smooth muscle cells [16]. Induction of many of the target genes of these transcriptional coactivators requires binding to the serum response factor [23, 24]. Caveolins and cavins, with exception for PTRF/CAVIN1, were in that study however unaffected by SRF knock down [16], arguing that they are controlled by the so called SAP domain. This domain is conserved in all myocardin family members [23] suggesting that all of them may harbor the potential to induce caveolae. An archetypal SAP domain-dependent target of MKL1 is tenascin C [43]. In contrast to caveolin-1/CAV1, which is an integral membrane protein, this protein resides in the extracellular matrix where it functions as an integrin ligand. Distinct localizations were indeed revealed in our co-staining experiments for TNC and caveolin-1/CAV1 which supported overall co-localization. It could be argued that coincident detection of nuclear MKL1 in the same cells would further strengthen the hypothesis that MKL1 was responsible for caveolin-1/CAV1 induction. An assessment of MKL1 distribution at a single time point is, however, a poor reflection of time-integrated MKL1 activity. In fact, distribution of a long-lived target protein, such as TNC, might be a better record of historical MKL1 activity.

Our studies on primary kidney epithelial cells demonstrated an MKL1-dependent induction of caveolins and cavins in culture. This finding is in good agreement with the work by Zhuang et al. [7], showing that while proximal tubular cells in situ are negative for these proteins, they are readily detectable when proximal tubules, or cells derived from them, are cultured in vitro. It has been previously demonstrated that cell dissociation, cell shape changes, and spreading cause MKL1 activation [35, 41]. Similar processes could be responsible for MKL1 activation and the following caveolin-1/CAV1 induction in the cultured kidney epithelial cells studied herein. MKL1 activity has also been shown to be modified by a number of coactivators and repressors. In cancer cells, MKL1 signaling has been shown to be inhibited by nuclear complex formation with SCAI, and in the original study a qPCR based tissue screen indicated high SCAI levels in the kidney [32]. We therefore hypothesized that SCAI may exert an inhibitory effect on MKL1 activity in proximal tubular cells. Furthermore, MKL1 signaling is intimately connected to the ratio of filamentous to globular actin. FLNA is a protein that functions to interconnect F-actin strands and also acts as a nuclear enhancer of MKL1 activity. Having noticed high levels of caveolar proteins in cultivated tubular cells, but absence of tubular expression in normal human kidney, we decided to analyze the presence of these modifying factors. We thus blotted for caveolin-1/CAV1, PTRF/CAVIN1, FLNA and SCAI in cortical tissue lysates from human kidney and lysates from primary tubular cells cultivated from the cortex of the same kidneys. Interestingly, FLNA was absent from cortical tissue lysates, but strongly induced in parallel with the increase in caveolin-1/CAV1 and PTRF/CAVIN1 expression in cultured proximal tubular cells. SCAI expression on the other hand virtually disappeared when the cortical cells were cultured. These results support the notion that MKL1 activity in tubular cells is enhanced by increased levels of FLNA and reduced expression of SCAI when cells are cultured in vitro.

We can rule out PEC or other contaminating cells as an explanation for increased levels of caveolar proteins in cultured kidney cortex cells. This is because caveolar protein induction was seen in small micro-dissected proximal tubular segments that should contain only proximal epithelial cells and no PECs. If increased levels of caveolar proteins were due to some rare cell, only a minor fraction of outgrowing cells would stain positive for caveolin-1/CAV1. When proximal tubules were manually isolated and cells dissociated, close to 100% of the resulting colonies were positive for caveolin-1/CAV1.

The induction of caveolar proteins has been suggested to occur through different mechanisms. Previous studies on the effects of hypoxia on caveolae have yielded contradictory results. Initial studies by Wang et al. [17] showed higher expression of caveolin-1/CAV1 in clear-cell renal cell carcinoma, where HIF1α and HIF2α are constitutively active. This was mimicked by hypoxia and was normalized by expression of the von Hippel Lindau (VHL) factor. A conserved hypoxia response element capable of binding HIF1α and HIF2α was moreover identified in the caveolin-1/CAV1 promoter, and VHL loss was associated with an increased density of caveolae. Together, these findings supported the view that caveolae are positively regulated by HIF. Regazzetti et al. [44] on the other hand found that hypoxia reduced cavin-1 and cavin-2 levels in adipocytes whereas caveolin-1/CAV1 levels remained unchanged. Hypoxia moreover reduced the density of caveolae at the ultrastructural level and these changes were mimicked by the HIF-1α inhibitor echinomycin. The apparently opposing results of these studies could be due to the use of different cells. We found, using primary kidney epithelial cells, that hypoxia reduced the mRNA levels of caveolin-2/CAV2 and SDPR/CAVIN2 whereas the caveolin-1/CAV1 level remained unchanged, in good agreement with the findings of Regazzetti et al. [44]. We conclude that hypoxia is unlikely to be a critical factor for induction of caveolar proteins in sclerotic kidneys.

A previous study has demonstrated that sublethal hydrogen peroxide concentrations up-regulate PTRF/CAVIN1 levels in human and mouse fibroblasts. H_2_O_2_ moreover increased association of caveolin-1/CAV1 with PTRF/CAVIN1 and increased the density of caveolae [22]. We speculated that this mechanism could be responsible for ectopic caveolin expression in sclerotic kidneys. We were however unable to induce caveolins and cavins using H_2_O_2_ despite testing different treatment protocols and close monitoring of cell viability. We cannot explain why fibroblasts and kidney epithelial cells respond differently to free radicals, but the proteomes of these cells are likely to be vastly different. The repressive effect of H_2_O_2_, especially on caveolin-2/CAV2, PTRF/CAVIN1 and SDPR/CAVIN2 protein levels and the lack of effect on caveolin-1/CAV1 protein, argued against a major role of oxidative stress in induction of caveolar proteins in kidney disease.

This study is not the first to document increased staining for caveolin-1/CAV1 in diseased proximal tubular epithelial cells. Vallés et al. [6] demonstrated increased proximal tubular staining for caveolin-1/CAV1 following longstanding ureteral obstruction. Caveolin-1/CAV1 induction has also been demonstrated in ischemic [3, 4] and toxic [5] kidney injury. Induction of caveolin-1/CAV1 thus appears to represent a conserved response of injured/regenerating proximal tubules. This may be a reflection of underlying MKL1 activation in regenerating epithelia. Recent work has established a role of MKL1 in kidney fibrosis [36, 42], but associated induction of caveolin-1/CAV1 was not demonstrated. The upstream mechanism of MKL1 activation in regenerating kidneys is presently unclear. Co-activation by FLNA and repression of SCAI, an inhibitor of MKL1 activity [32] that has recently been demonstrated to play a role in kidney fibrosis [42], are attractive possibilities.

## Conclusions

Our results show induction of caveolin and cavin expression in sclerotic kidneys and in kidney epithelial cells in culture. In vitro induction of caveolin-1/CAV1 can be inhibited and mimicked by MKL1 inhibition and overexpression, respectively. Caveolin-1/CAV1 also co-localizes with the archetypal MKL1 target tenascin C in both healthy and sclerotic kidneys, arguing for a similar transcriptional mechanism in situ. Our findings provide further support for the notion [16] that the myocardin family members may explain the tissue distribution of caveolae also in the kidney. MKL1 mediated induction of tubular caveolae during renal disease most probably plays an important role in the renal response to injury, requiring further study.

## Additional files

Additional file 1: Figure S1. Negative control for Immunohistochemical stainings. Immunohistochemical staining of human renal tissue, with exclusion of primary antibody incubation step, serving as a negative control for stainings presented in Fig. 1. G - glomerulus, PT - proximal tubules, SMC – smooth muscle cells, PEC - parietal epithelial cells. (TIFF 3675 kb)

Additional file 2: Figure S2. Validation of the protocol for manual isolation of proximal tubules. Tubules isolated according to size and refractive properties were immunohistochemically stained or incubated with segment specific lectins. In A staining of isolated proximal tubules with the proximal marker CD10 is shown, whereas the distal marker E-cadherin stains negative (B). Tubules were also stained with fluorescein labeled tubular segment specific lectins: Positive staining results after incubating proximal tubules with the proximal tubule marker *Lotus tetragonolobus* Lectin (LTL) is shown in (C). In D the staining of distal tubules by the distal marker *Dolichos biflorus* Agglutinin (DBA) is shown. (TIFF 5794 kb)

Additional file 3: Figure S3. Quantification of Western blots in Fig. 5C for caveolin-1/CAV1, caveolin-2.CAV2, caveolin-3/CAV3, PTRF/CAVIN1, SDPR/CAVIN2 and PRKCDBP/CAVIN3 as compared to HSP90 in kidney epithelial cultures of 50 or 100% confluency or 3 and 5 days post confluency (p.c). *n* = 3, *(*p* < 0.05), **(*p* < 0.01), ***(*p* < 0.001). (TIFF 789 kb)
