# Supplementary figures and images for: Injury induced expression of caveolar proteins in human kidney tubules - role of megakaryoblastic leukemia 1
Source: BMC Nephrol. 2017 Oct 24;18:320. doi: 10.1186/s12882-017-0738-8 (PMC5655893; doi:10.1186/s12882-017-0738-8)

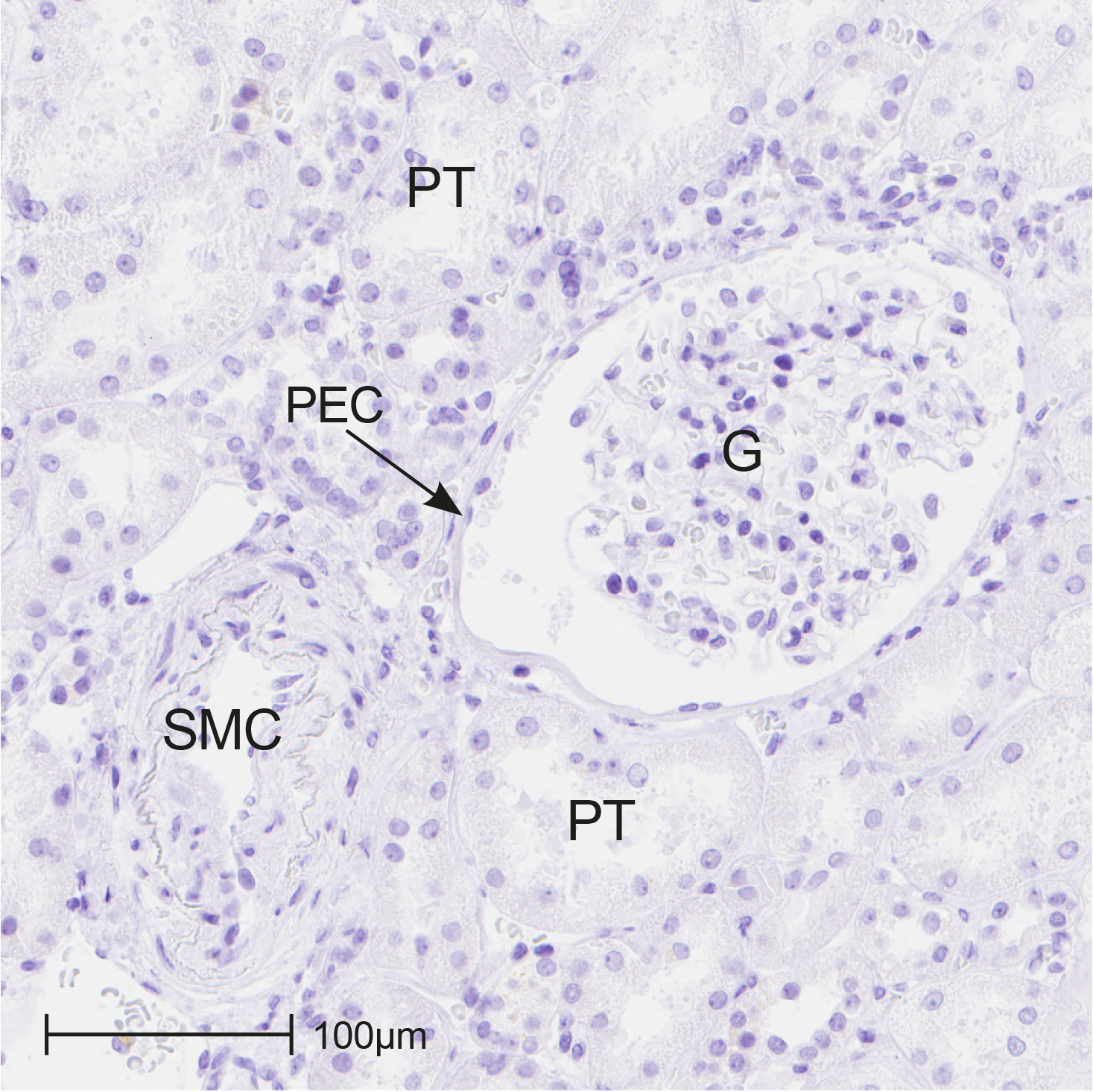

Supplement: Supplementary file 1 — Negative control for Immunohistochemical stainings. Immunohistochemical staining of human renal tissue, with exclusion of primary antibody incubation step, serving as a negative control for stainings presented in Fig. 1. G - glomerulus, PT - proximal tubules, SMC – smooth muscle cells, PEC - parietal epithelial cells. (TIFF 3675 kb) [file 12882_2017_738_MOESM1_ESM.tif]

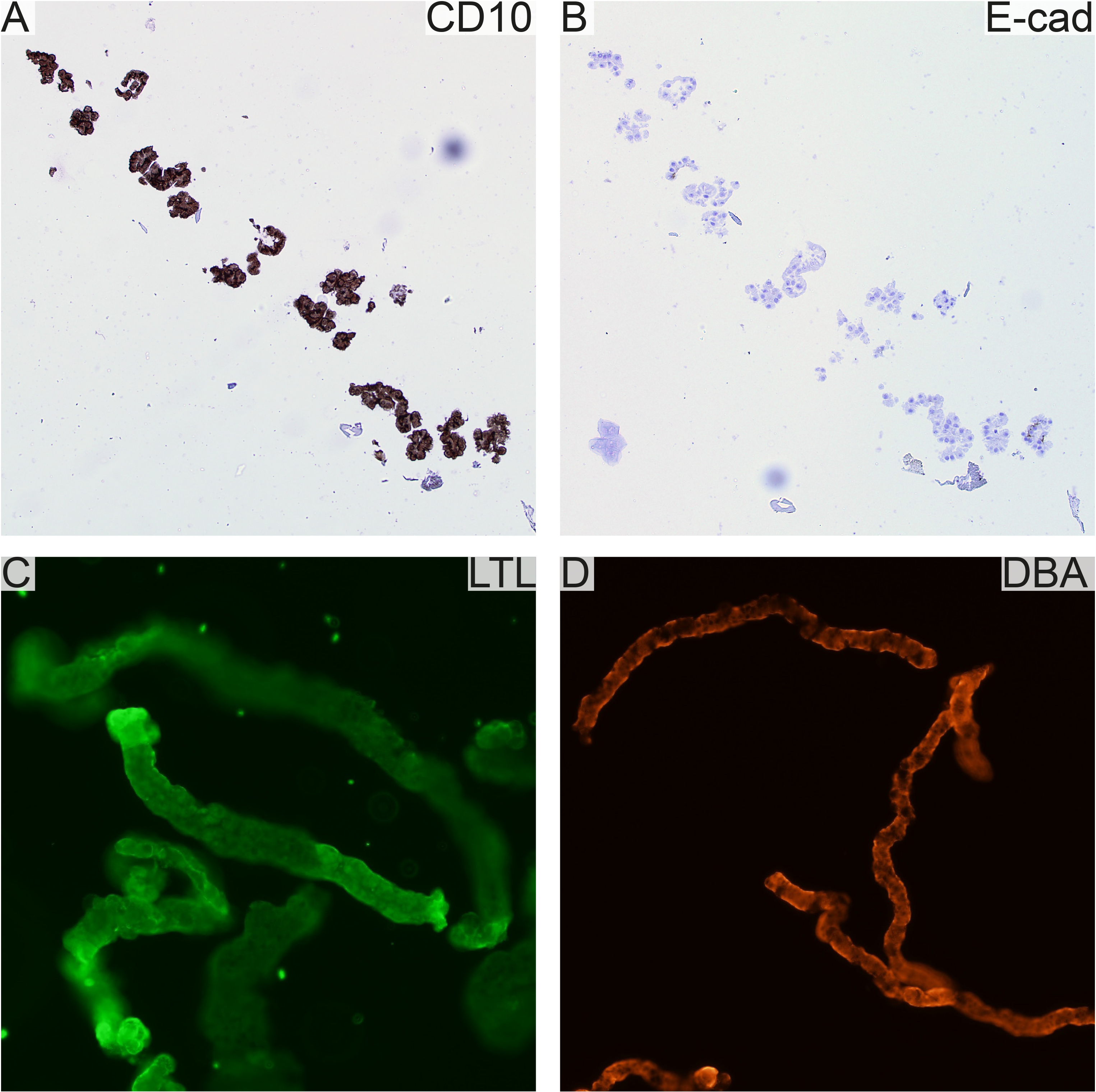

Supplement: Supplementary file 2 — Validation of the protocol for manual isolation of proximal tubules. Tubules isolated according to size and refractive properties were immunohistochemically stained or incubated with segment specific lectins. In A staining of isolated proximal tubules with the proximal marker CD10 is shown, whereas the distal marker E-cadherin stains negative (B). Tubules were also stained with fluorescein labeled tubular segment specific lectins: Positive staining results after incubating proximal tubules with the proximal tubule marker Lotus tetragonolobus Lectin (LTL) is shown in (C). In D the staining of distal tubules by the distal marker Dolichos biflorus Agglutinin (DBA) is shown. (TIFF 5794 kb) [file 12882_2017_738_MOESM2_ESM.tif]

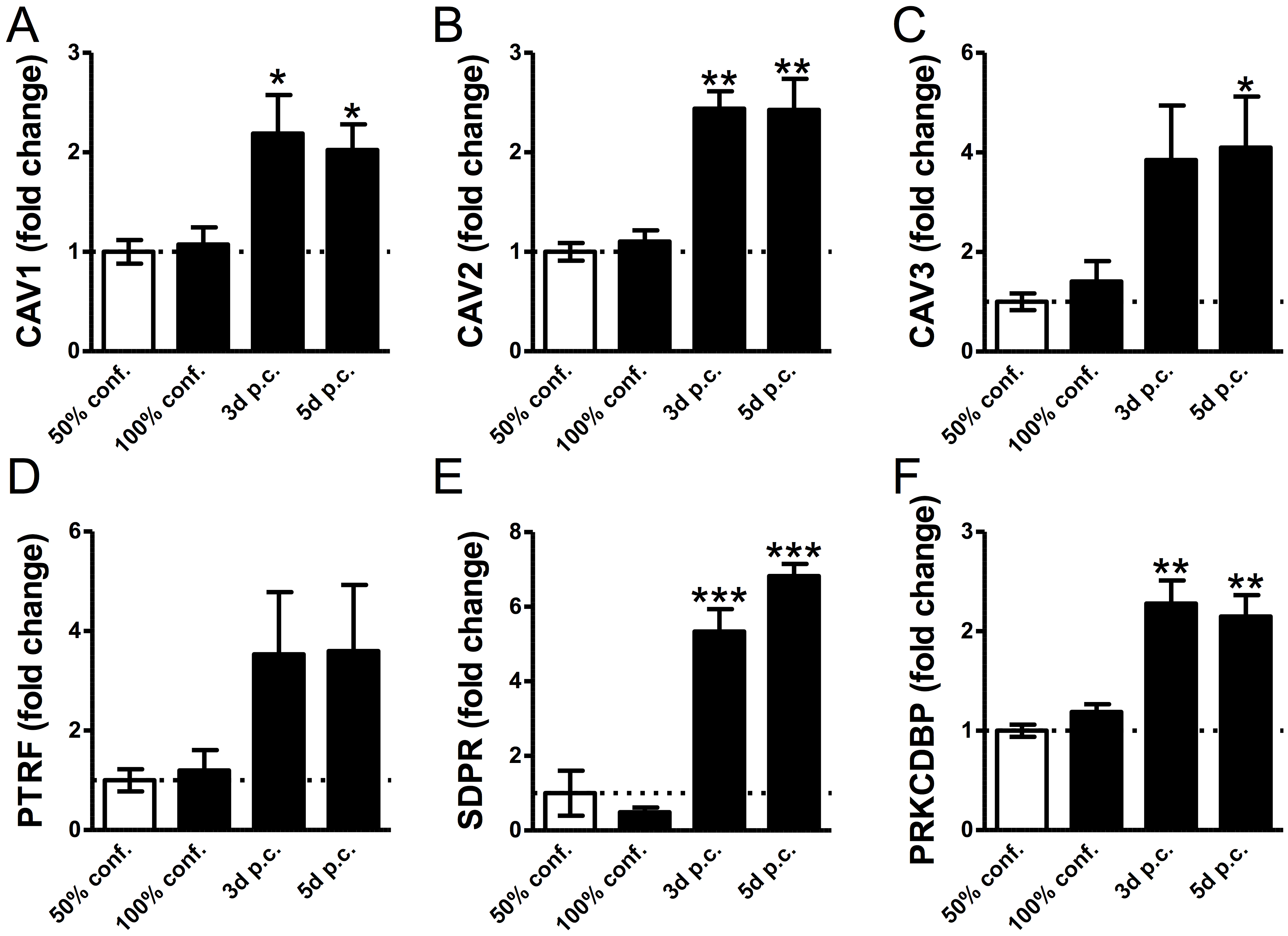

Supplement: Supplementary file 3 — Quantification of Western blots in Fig. 5C for caveolin-1/CAV1, caveolin-2.CAV2, caveolin-3/CAV3, PTRF/CAVIN1, SDPR/CAVIN2 and PRKCDBP/CAVIN3 as compared to HSP90 in kidney epithelial cultures of 50 or 100% confluency or 3 and 5 days post confluency (p.c). n = 3, *(p < 0.05), **(p < 0.01), ***(p < 0.001). (TIFF 789 kb) [file 12882_2017_738_MOESM3_ESM.tiff]
